# Robust language-based mental health assessments in time and space through social media

**DOI:** 10.1038/s41746-024-01100-0

**Published:** 2024-05-02

**Authors:** Siddharth Mangalik, Johannes C. Eichstaedt, Salvatore Giorgi, Jihu Mun, Farhan Ahmed, Gilvir Gill, Adithya V. Ganesan, Shashanka Subrahmanya, Nikita Soni, Sean A. P. Clouston, H. Andrew Schwartz

**Affiliations:** 1 Department of Computer Science, Stony Brook University, Stony Brook, NY USA; 2 Department of Psychology, Stanford University, Stanford, CA USA; 3 Institute for Human-Centered A.I., Stanford University, Stanford, CA USA; 4 Department of Computer and Information Science, University of Pennsylvania, Philadelphia, USA; 5 Department of Family, Population, and Preventive Medicine, Renaissance School of Medicine, Stony Brook University, Stony Brook, NY USA

**Keywords:** Interdisciplinary studies, Psychology

## Abstract

In the most comprehensive population surveys, mental health is only broadly captured through questionnaires asking about “mentally unhealthy days” or feelings of “sadness.” Further, population mental health estimates are predominantly consolidated to yearly estimates at the state level, which is considerably coarser than the best estimates of physical health. Through the large-scale analysis of social media, robust estimation of population mental health is feasible at finer resolutions. In this study, we created a pipeline that used ~1 billion Tweets from 2 million geo-located users to estimate mental health levels and changes for depression and anxiety, the two leading mental health conditions. Language-based mental health assessments (LBMHAs) had substantially higher levels of reliability across space and time than available survey measures. This work presents reliable assessments of depression and anxiety down to the county-weeks level. Where surveys were available, we found moderate to strong associations between the LBMHAs and survey scores for multiple levels of granularity, from the national level down to weekly county measurements (fixed effects *β* = *0.34* to *1.82*; *p* < 0.001). LBMHAs demonstrated temporal validity, showing clear absolute increases after a list of major societal events (+23% absolute change for depression assessments). LBMHAs showed improved external validity, evidenced by stronger correlations with measures of health and socioeconomic status than population surveys. This study shows that the careful aggregation of social media data yields spatiotemporal estimates of population mental health that exceed the granularity achievable by existing population surveys, and does so with generally greater reliability and validity.

## Introduction

Mental health is an important public health concern, causing economic impact and loss of quality of life. Recent estimates suggest that depression affects 19.4 million Americans (7.8% of the population, 2020 est.) each year^1^, while generalized anxiety disorder affects ~6% of the US population (19.8 million people, 2010 est.)^2^. Globally, mental health conditions are the fifth-most common cause of reduced quality of life^3^. Critically, poor mental health is thought to play a central role driving recent increases in prevalence and severity of “deaths of despair”^4,5^ in part due to the influence of poorer mental health on suicide attempts and suicide mortality obesity^6^, and opioid-related overdoses^7,8^.

Public health researchers and policymakers seek to understand and actively respond to emerging and changing conditions^9–11^. Yet, current standards for monitoring mental health outcomes rely on subjective survey responses that have limited temporal or regional resolution. For example, yearly changes in depression are measured only by annual Gallup polling^12^ and a handful of national surveys^13^ while anxiety is not regularly assessed in any of these surveys^14^. Other works have predicted population health statistics like mortality^15^, well-being^16,17^, substance use^18^, viral outbreaks^19^, smoking^20^, obesity, and flu^21^ using Twitter language from a limited number of counties^20^ or at the state level^21^. Nevertheless, improving geospatial resolution can provide researchers with tools to more reliably assess the distribution^22^ and determinants of disease^23^. Similarly, a wealth of small studies using ecological momentary assessment suggest that observations made on shorter timescales routinely identify symptoms and correlates that are otherwise inaccessible to researchers^24,25^.

Applying validated measures of depression and anxiety, assessed objectively at regular time intervals at the county level could transform research in population mental health, allowing researchers to locate clusters and reasons for changes to poorer mental health^26^. All such assessments are not direct measurements of mental health incidence, which can only be done in a clinical setting. Nevertheless, measures that are convergent with subjective experiences are an invaluable resource for population health researchers. Since originally proposed, language-based assessments have developed to become a flexible source of observed emotions and behaviors from individuals^27^, often with greater accuracy and predictive power than existing survey-based measures^28^. Further, recent work has found significant increases in convergent validity via post-stratification techniques^29^ to address known selection biases^30,31^.

**Fig. 1:**
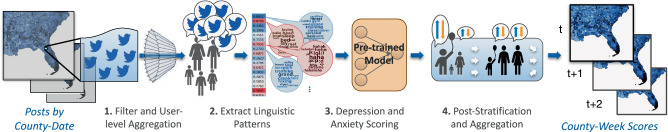
The Language Based Mental Health Assessment pipeline. Visual overview of the *language-based mental health assessments* pipeline. County-mapped messages are filtered to self-written posts, from which language features are extracted and passed through pretrained language-based mental health assessments to generate user scores. These scores are then reweighted to better represent county demographics and are then aggregated to communities in time.

Here, we integrated a series of recent advances into a single pipeline capable of generating *language-based mental health assessments* (*LBMHAs*: Fig. 1), to produce appraisals of anxiety and depression over regions and time. Our process of evaluation follows modern psychometric principles^32^, covering key steps from the long-discussed and studied topic of determining the efficacy of psychological construct measurement^33–35^. Modern psychometric principles recommend that psychological assessments (that measure something inherently latent) undergo a series of evaluations for both *reliability* and *validity*. The idea is that no single evaluation can yield - on its own - a judgment about the quality of the psychological assessment. “The entity that the [assessment] is measuring is normally not measurable directly, and we are only able to evaluate its usefulness by looking at the relationship between the test and the various phenomena that theory predicts”^32,pg. 44]^. Key concepts for such evaluations are formalized as reliability and validity. *Reliability* is concerned with evaluating the consistency or precision of a measure, capturing if the measure is self-consistent across multiple retests or internal samplings (within an expected margin of error). *Validity* is concerned broadly with accuracy. It spans different dimensions, including convergent and external criterion validity. *Convergent validity* measures the extent to which the measure agrees with an accepted measure (this is in line with what is often referred to as prediction accuracy in machine learning contexts), while *external criteria* capture the extent to which the measure predicts external outcomes (such as behaviors) that the measured construct is expected to be associated with.

Our overarching study goal was to examine whether LBMHAs could monitor population mental health with reliability and validity. Following psychometric principles, we first evaluated the reliability of LBMHAs contrasted with standard surveys. We then examined reliability over varying time and space units (from annual to daily and national to townships) ultimately setting minimum observational thresholds. Next, we evaluated the convergent- and external-validity of the LBMHAs as compared to the most extensively collected mental health-related surveys available for the same time period (Gallup’s COVID-19 Panel), both cross-sectionally and longitudinally. Finally, to facilitate open scientific inquiry, we released the LBMHA measurements as well as documentation and an open-source toolkit for independently deriving mental health estimates.

## Results

**Fig. 2:**
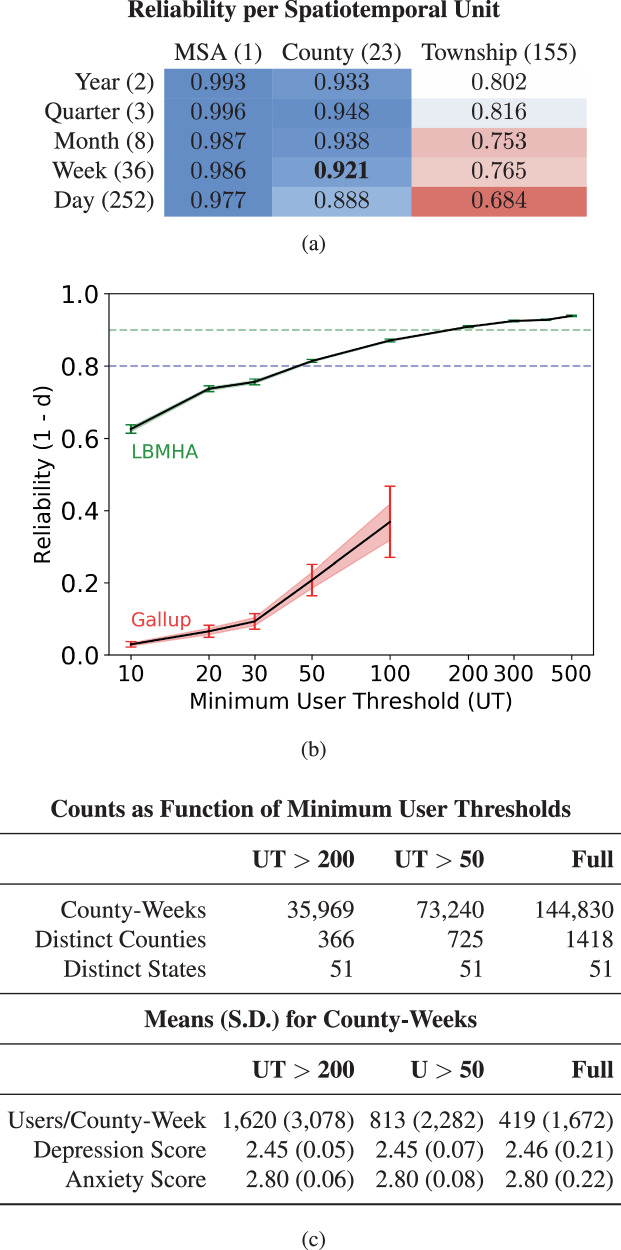
Reliability-informed thresholding. Spatiotemporal reliability of language-based mental health assessments of depression across different granularities of space and time in the New York metropolitan area. The heatmap in (**a**) shows the 1 − Cohen’s *d* reliability of select New York metropolitan depression data, at each space and time unit ≥20 unique users were required. From this heatmap, we target the smallest time unit from the smallest space unit greater than 0.9, which is county-week. The plot in (**b**) shows how the reliability of a county-week measurement of depression increases with the minimum number of unique users required to consider that county-week. In the case of Gallup data, after a UT of 100 none of the county measurements can meet the minimum criteria to be reported. Horizontal lines are drawn at 0.8 and 0.9 reliability, which were used to select a 50 and a 200 county user threshold. The standard error of the reliability is shown with red shading, and the 95% confidence interval is shown with error bars. The county-year Intraclass Correlations, test-length corrected (ICC2^82^;) at a UT of 50 are *ICC*2 = 0.33 for Gallup Sadness and *ICC*2 = 0.97 for LBMHA depression, while at a UT of 200 are *ICC*2 = 0.87 for Gallup and *ICC*2 = 0.99 for LBMHA. **c** shows data descriptives for the county-week dataset after applying a user threshold of 50 and 200 as per the reliability findings and applying all other thresholds.

**Fig. 3:**
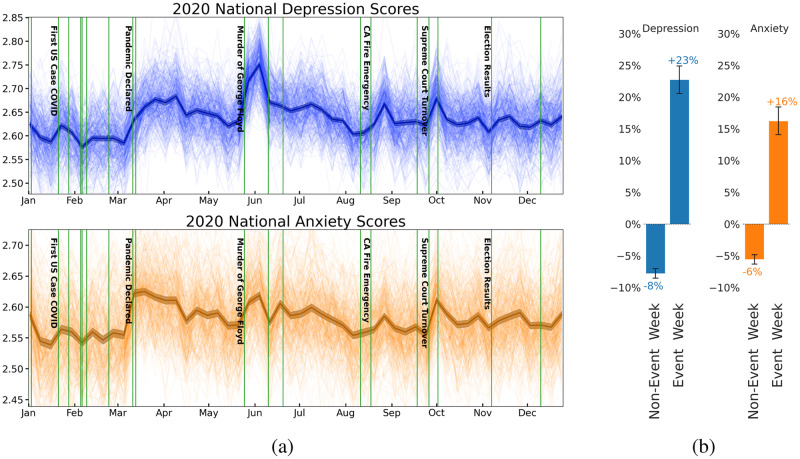
Main measurements and effects of major events. Shown in (**a**) are depression (blue) and anxiety (orange) measured at the nation-week level for all of 2020, controlling for 2019 measurements. All scores shown are based on aggregated user scores that are scaled from 0 to 5, with 5 representing the highest level of depression/anxiety. Labeled green vertical markers are placed at the start of major events. In dark blue/orange, we have plotted nation-week averages alongside 95% confidence intervals, and in thinner lines, we show similar trends for individual counties. This figure requires counties to contain at least 200 unique (UT = 200) users in a given week to be included, this gives 370 distinct counties spanning the year 2020. **b** contains an analysis of the impact of weeks containing major US events against weeks without similar events. Shown are the z-scored percent differences from the prior week in LBMHAs between weeks that do contain major US events and those weeks that do not. Confidence interval bars are generated from Monte Carlo bootstrapping on 10,000 samples from the pool of either event weeks or non-event weeks and re-calculating mean z-scored percent differences between the drawn samples.

Assessments have been generated for all counties that demonstrated sufficient posting history to be considered reliable per the thresholds determined in the reliability portion of this work as found in Fig. 2. The nation-week depression and anxiety scores from our language-based mental health assessments in 2020 adjusting for 2019 can be found in Fig. 3. The results as shown cover all weeks in 2020, and depict the included counties alongside the national average result in bold. For this visualization, a county must have at least 200 unique users in a given week to be included.

### Reliability of spatio-temporal resolutions

Figure 2 shows the relationship between different resolutions of time and space on the split-half reliability of our measurements. Underlying all measurements we use depression scores within the given spatio-temporal cohorts. The threshold (Cohen’s *d* = 0.1) was crossed for all township-level measurements, all but one county-level measurement, and all of the MSA-level measurements. Looking across time for counties we determine that the week level is the smallest time resolution with our smallest accepted space resolution to have a reliability (1 − Cohen’s *d*) that is ≥0.9. Using this county-week finding, we observed that once there were at least 50 users (user threshold [UT] = 50) reliability exceeded 0.8. In this context, the UT can be understood as the minimum number of unique users required by a county to be included in our analysis. At a UT of 200, it is possible to obtain a reliability measurement of 0.9 indicating no effect. This analysis led us to create standard county-week threshold guidelines at UT of 50 and 200. The use of a 50 UT (725 distinct counties) reflects the highest number of counties that are directly usable versus the more restrictive 200 UT (366 distinct counties).

### Convergent Validity

**Fig. 4:**
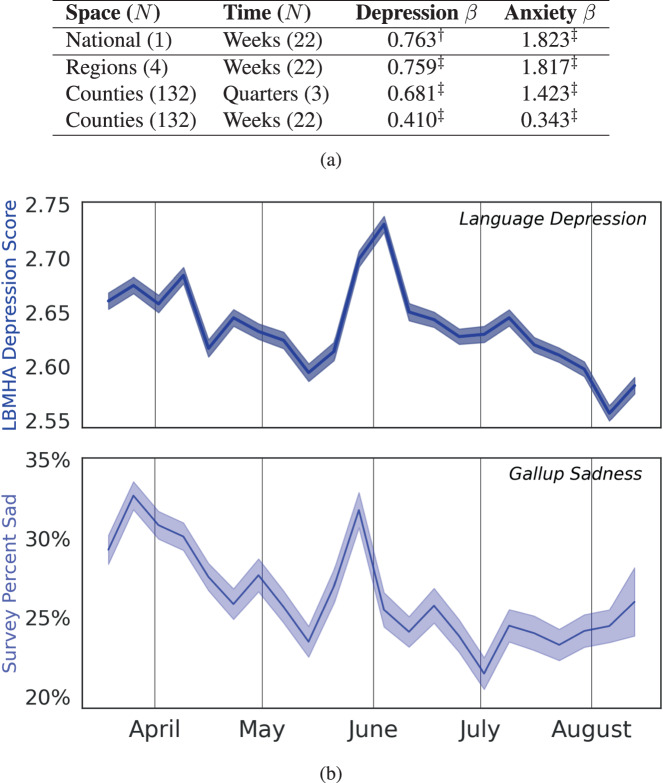
Convergent validity. Convergent validity between language-based mental health assessments and survey-based measures longitudinally at different spatial resolutions. **a** shows fixed-effects coefficients between language-based mental health assessments (LBMHAs) and Gallup COVID-19 Panel Questionnaire measurements. Depression *β* compares our language-based depression scores to Gallup’s surveyed sadness scores via hierarchical linear modeling coefficients. Anxiety *β* compares our language-based anxiety scores to Gallup’s worry scores. **b** shows the national plots of depression as measured by LBMHAs and sadness as measured by Gallup. Both Questionnaire and LBMHA measures are held to reliability constraints as described in our section on reliability. Between the two national-week plots shown, there is a *β* = 0.763. Results significant at: ^‡^*p* < 0.001, ^†^*p* < 0.01.

Figure 4 depicts the outcomes of our multi-level fixed effects model between Gallup’s self-reported sadness and worry against our language-based assessments of depression and anxiety. At all levels evaluated for fixed effects, we find our *t*-test *p*-value to be significant to 0.01. At the nation-week level, we find that the survey and language are correlated (Pearson’s *r* = 0.34) with depression and sadness, and anxiety and worry (Pearson’s *r* = 0.67). Fixed-effects coefficients between survey and language findings indicate higher agreement in analyses using larger spatial and temporal units, with the highest coefficients coming from a national-week analysis (see Supplementary Fig. 6 for a classification interpretation of this problem). At finer resolutions, we nevertheless still identify statistically significant positive values leading us to conclude that county-week level measurements may reflect greater local sensitivity that might not as consistently correspond to the greater national trends. Further, the robustness analyses in panel (**a**) suggest the post-stratification is leading to a minor improvement at the national-week level (see Supplementary Fig. 5 for a robustness evaluation of these findings relative to spatial auto-correlation).

### External criteria

**Fig. 5:**
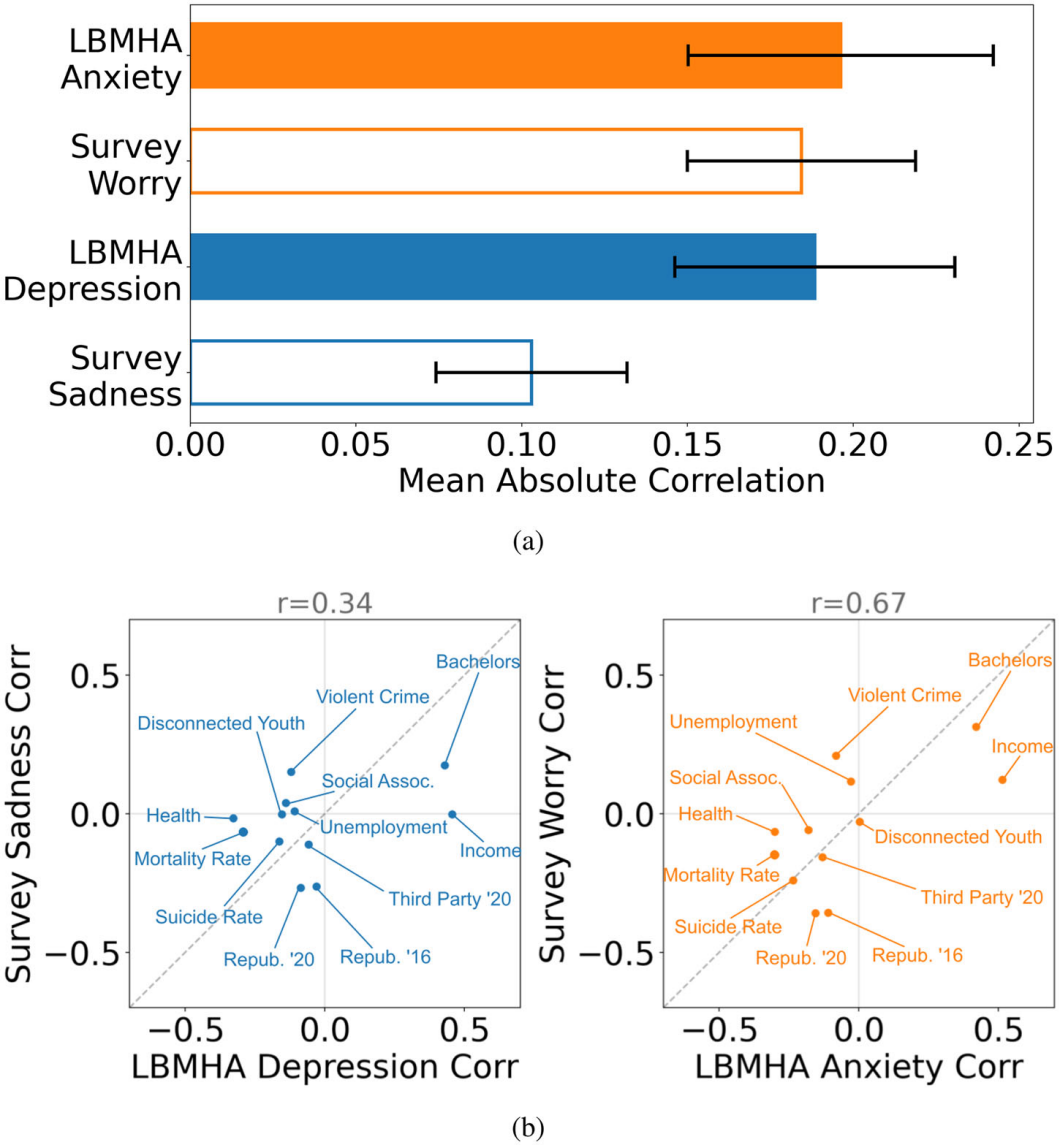
External validity. Cross-sectional associations between LBMHAs of Anxiety/Depression and survey assessments of Worry/Sadness against external criteria from Political, Economic, Social, and Health (PESH) variables across *N* = 262 counties. **a** compares the average absolute effect Pearson correlations of LBMHA and Survey measures against external PESH variables. **b** shows scatterplots of correlations between external criteria and our LBMHAs on one axis and the surveyed results on the other axis. All counties included meet our reliability requirements. Perfect agreement is shown as a diagonal dashed line. The association is measured using Pearson correlation. For the PESH variables examined we observe a Pearson correlation of Pearson correlations of 0.67 for Anxiety-Worry and 0.34 for Depression-Sadness, both findings are significant to *p* < 0.01.

In Fig. 5 we graphically represent the validity of our measures against other established county measures. The source of external county-level data is the County Health Rankings^36^ which track PESH (Political, Economic, Societal, and Health) outcomes on a county-year scale. We observe strong agreement between the correlations of our LBMHA scores and the Gallup self-reported results with these PESH variables.

In Fig. 3b we examine the difference between event weeks and non-event weeks. We find an increase on average of the mean absolute difference of both depression (23%) and anxiety (16%) during weeks in which major US events occur. Likewise, we see a “resetting" effect wherein non-event weeks on average decrease the general level of both anxiety (6%) and depression (8%), however nationally across 2020 the absolute unadjusted level of both measures is increasing. These results over a comparison of event and non-event weeks for several counties suggest that changes in community mental health can be attributed to specific events.

### American communities comparison

**Fig. 6:**
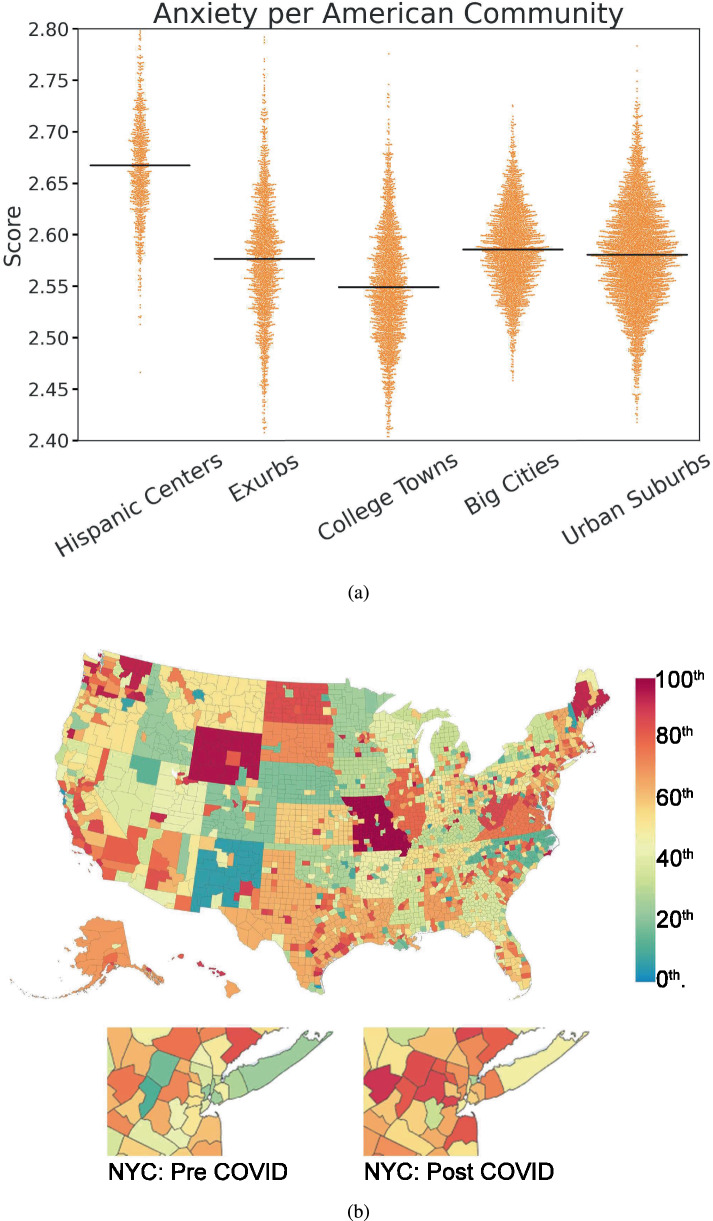
Community-level analysis of Language Based Mental Health Assessments. Scores within communities in 2020 and county-mapped anxiety before and after COVID-19 is declared a pandemic. In (**a**) we showed the 5 community types most commonly represented in our data, out of 15 possible communities as defined by the American Communities Project, are shown in order by the number of measurements captured. A black horizontal mean line is overlaid on swarm plots of the county-week measurements for each community type. In (**b**) percentile county-level measurements of anxiety are shown, where red shows where anxiety is highest and blue where anxiety is lowest. Pre-declaration is defined as two months before the COVID-19 National Emergency declaration (3/13/2020) and post-declaration is defined as two months after the declaration. The top section depicts national anxiety per county in the post-declaration time window, while the bottom section shows a zoomed-in view of the NYC Metropolitan Area in each time window. Super-county binning is performed to report results for counties that are not individually reliable.

Figure 6a shows how anxiety differs across American community types. We select the five communities for which we have the greatest representation in our final dataset of county-week LBMHAs. We observe that the Exurbs, defined as communities that “lie on the fringe of major metro areas in the spaces between suburban and rural America”, score as the most anxious and most depressed of observed U.S. communities. Although the overall difference between community types is modest, we anticipate that examinations of factorized measures of anxiety and depression may show larger discrepancies.

## Discussion

Anxiety and depression are costly, under-diagnosed and under-treated, and, while common overall their prevalence varies across time and location. For example, depression alone has been attributed as the second highest mechanism for loss of disability-adjusted life years, more than cancer and diabetes^37^. The present study used 15.3 billion words from 2.2 million people living across the U.S. to evaluate the potential of using a modern approach for measuring public mental health, from behavioral patterns (language use). Notably, we were interested in whether this approach could produce epidemiologically valid and reliable scores that could be used to understand variability by geography and change in public mental health over time. We found this approach achieved much greater regional and temporal resolution (e.g., within U.S. counties each week) while also achieving high convergent validity for the limited amount of high-resolution survey-based assessments available.

We put together many recent developments in the best practices for social media-based well-being assessments. First, we utilized the notion of a digital cohort, whereby documents are aggregated through people, mirroring modern surveys^38^. Then, we utilized new computational methods to mitigate epidemiological selection biases using *robust poststratification*^29^. Additionally, we applied domain adaptation techniques to our lexica which have been shown to result in meaningful performance gains^39^. We also contributed an analysis of the statistical reliability of LBMHAs in order to establish minimal sampling thresholds. Finally building on epidemiological work we controlled for seasonality effects by adjusting using previous data to find the changes attributable to events occurring in 2020^40^.

Our LBMHA pipeline reported similar temporal patterns, both nationally and at the county level, to existing U.S. weekly data from Gallup, while also demonstrating the ability to report reliable results for a far larger number of counties and weeks (see Supplementary Figs. 1 and 2). Further, LBMHAs captured changes in depression and generalized anxiety that corresponded to major events in 2020, including those of the COVID-19 pandemic declaration.

Symptom presence and severity cannot be readily measured for mental illness because, unlike physical illnesses, they have no highly sensitive biomarkers. Furthermore, self-reports are suspected to be hindered by stigmatization associated with mental illness. This work improves the assessment process by joining recent research focused on identifying biobehavioral measures indicating the presence of mental health disorders including, for example, functional^41^ and structural neuroimaging^42^ as well as those cellular changes^43^. However, the present study signifies a shift in thinking by changing focus away from putative biomarkers for behavioral disorders toward focus on determining levels of depression and anxiety by observing individuals’ natural unedited communication behaviors.

The shared geographic and temporal resolution presented in this study could enable the ability to understand the role of social, economic, or natural events and mental health at unprecedented resolutions. This study shows that improved resolution of mental health outcomes reflects the presence of major national events. For example, following the murder of George Floyd, language-estimated depression prevalence showed a clear increase, mirroring similar trends observed in Gallup survey data^44^.

COVID-19 first arrived in the U.S. during the data collection period (2019–2020). Consistent with prior research, we found that COVID-19 caused a rapid increase in depressive symptoms and generalized anxiety across the U.S. that did not dissipate before 2021. The distribution of poorer mental health was widespread and included large increases in regions with relatively low pre-pandemic levels of depression and anxiety. For example, the average level of anxiety increased from the lowest to the highest levels in Kansas in the months after the pandemic. These mental health shocks also began late in 2019, when COVID-19 was first identified globally and spiked in early March 2020 when much of the Northeastern U.S. was shuttered and people in open states chose to self-isolate. While these effects show the value of the approach for understanding how public mental health changes in a pandemic, these data also show that anxiety and depressive symptoms had not yet returned to pre-pandemic norms by the end of the observational window.

As with any social media platform, many users will self-project—attempt to behave in ways that influence how others perceive them. In CTLB that would manifest as posts that emphasize the positive qualities of the author. However, our language-based assessments treat language use as a behavior and do not rely on a priori assumptions about positive language signaling positive psychological traits. Rather, the LBMHAs we used are data-driven. Past work has shown that social media language behavior, whether motivated by self-presentation or not, is predictive of standard non-NLP measures of psychological attributes such as depression questionnaires^45,46^, personality tests^47–49^, annotated mental health self-disclosures^50^, medical depression diagnoses^15^, or public health surveys^20^.

We observed mental health using posts from geo-located Twitter users, as this allowed us to examine rapid changes in mental health at scale. LBMHAs have been reliably used outside of social media. For example, studies of psychological stress have noted that LBMHAs can aid in identifying individuals with at risk of poorer postpartum mental health when relying on mothers’ diaries^51^ and for identifying poorer long-term prognosis in post-traumatic stress disorder when relying on oral histories^28^.

Results from this study should be interpreted in light of a number of limitations. First, many U.S. counties with small populations or a small number of social media users had to be combined into super-counties to provide reliable estimates. Accounting for only a small percentage of the total US population, these are regions that are often under-represented in research studies. This approach allowed for their inclusion but nevertheless resulted in units covering large geographic areas. Second, no Twitter pipeline can fully remove non-human users. However, the pipeline’s organization is suited to mitigating the influence of social bots^52^. The exclusion of tweets with hyperlinks, retweet, and duplicates in particular emphasizes genuine thoughts expressed by humans rather than social bots which have been seen to generate far less novel content, focusing on retweeting and making posts with URLs^53,54^. Further, our work analyzes user aggregate measures which serve to minimize the effects of individual accounts that might post at higher rates than other users. This hierarchical aggregation has improved outcomes in previous works^55^ where aggregating through users was found to remove the effects of accounts that posted at non-human rates.

While this evaluation brings together steps that were validated on different sets of data, what we present here only covers 2019 and 2020 in the United States. Using 2019 as a control addresses some effects of having a short time-frame, such as seasonal effects. However, language evolves over time. Social media has a so-called “semantic drift” whereby words slowly begin to take on differing meanings^56–58^. Thus, analyses of LBMHAs in future years or in different countries should include convergent validations, reliability testing, and potentially apply further model adaptations. Of note, the protocol described in this work was not preregistered, but this study utilized two pre-trained models and not models we fit as part of this work. Additionally, social media platforms aren’t rigid organizations and can change ownership, policies, and user populations. Twitter recently changed ownership resulting in new content moderation strategies and data sharing practices. While other sources of public language exist, such as Mastodon or Reddit, the evaluations of this paper are focused on prior years of Twitter, and any application after the recent ownership change or to other platforms requires further validation. We position this work as part of the larger advocacy for increasing open data availability for non-profit researchers working on population health studies using social media data, and we encourage future evaluations of the LBMHA pipeline and pre-trained models on different years and different platforms of data.

This work utilized lexicon-based models (i.e., weighted dictionaries). Recent work has shown that transformer-based language models (i.e., those used by programs like ChatGPT) can result in performance gains in assessing mental health from language^59,60^. Lexical models had two main advantages when we began this project: First, they have a longer history of use and the models we used have been through a wider range of validations at the person-level^61,62^. Second, they are much faster to run, requiring much fewer computing resources than large language models. As large language models (LLMs) become further validated at the person-level and more efficient to run across billions of texts, we anticipate that LBMHAs will begin to utilize them. We would expect LLM approaches to implicitly handle semantic drift and other word-context issues. The completion of this work supports future pipelines that can be recreated with transformer-based models.

While this work used a data-driven approach for mapping language use to mental health assessments it does not include an evaluation of how individual posted words on social media are indicative of clinical depression or anxiety. We cannot determine whether a person who uses depressive language actually meets diagnostic criteria for depression and therefore it remains unclear whether increases in language as noted here convert into increases in diagnoses of depression or generalized anxiety. Future studies will be required to evaluate these individual posted words as instruments for measuring clinical mental health.

The strength of this epidemiological study is that it applied scalable methods meant to improve generalizability on a sample that included nearly 1 billion observations on 2 million individuals (0.6% of the U.S. population) across more than 1400 U.S. counties. These results validate temporal results previously derived from U.S. polling sites interested in tracking mental health.

To date, most efforts to profile the mental health of people in the U.S. and globally rely on subjective responses to survey prompts. These surveys may be biased by the tendency for people to under-report less desirable or stigmatized traits, such as the presence of mental illness. Up to date access to objective measures of changing mental health could improve the ability to allocate scarce mental health treatment resources in a time of great need and will facilitate new analyses that can help us to better understand the risk factors and consequences of depression and anxiety in population health.

This work lays a foundation to expand on the AI-based population assessment process to both refine the tools and improve the generalizability of assessments as we move this work into public mental health monitoring programs. Furthermore, quasi-experimental designs using rich temporal data have shown potential in revealing deeper facets of longitudinal effects suffered by those struggling with depression^63^. Such assessments over social media also lend themselves to integration into social network effects. The relationships between local social graphs and the potentially observed assortativity of mental health phenomena (be it through contagion, selection, or some other mechanism) are complex and were beyond the scope of the current article.

Going beyond population health, language-based mental health assessments from social media have applications in the localized mental health of educational, professional, and medical organizations^64,65^. For example, integrating a system using the pipeline described here into an opt-in program for communications platforms for high burnout professions, such as hospitals, WHO employees, or legal offices. This study suggests that the careful analysis and aggregation of social media data can yield spatiotemporal estimates of population mental health that exceed surveys in resolution and potentially in reliability and validity.

## Methods

### Data

As our main source of social media data, we introduce an updated version of the original *County Tweet Lexical Bank, CTLB*^38^ which we refer to as *CTLB-19-20*. This new version contains a cohort of county-mapped Twitter (now *X*) posts from accounts, spanning 2019 to 2020. Following county-mapping procedures of ref. ^16^, these county-user pairs were derived from posts with either explicit longitude/latitude pairs or the first instance of a self-reported user location in the account public profile. The location string was previously found to be 93% accurate compared to human assessments^16^. While techniques exist for locating users within kilometers^66,67^, we opt to not use these methods as they introduce a confounder by using language for both location inference and assessments. Previous works have also shown convergent validity with other related outcomes demonstrating the robustness of language-based assessments despite the presence of potential noise in the geotagged data^68,69^.

The unprocessed CTLB-19-20 contained 2.7 billion total posts from a cohort of 2.6 million users over 2019 and 2020, after filtering this would result in ~1 billion posts from 2.2 million users (see Table 1). For each post in this dataset, we retain the date it was posted, a unique user identifier, the original text body, and the US county that the poster is from.

**Table 1:** Coverage included in the filtered County Tweet Lexical Bank dataset from 2019 to 2020

CTLB Data Descriptives	
	Count
Word Instances	15,361,519,145
Posts	992,194,052
Unique Words	57,448,057
Users	2,198,980
Counties	1490

	Mean (S.D.)
Posts per User	451.2 (749.9)
Posts per User/Week	10.2 (25.7)
Users per County	1249.4 (4,609.7)

Filtering consisted of excluding non-English posts, reposts, posts containing a hyperlink, and duplicated posts from users. Standard deviations are included in parentheses next to mean measurements.

### Filter and people aggregation

Following ref. ^38^, preprocessing steps filtered out non-original content, which has been shown to increase the accuracy of social media-based population assessments^27^. Posts are only included if they are marked likely to be English according to the langid package^70^, and then they are further filtered to remove retweets, posts containing hyperlinks (i.e., posts likely of non-original content), and finally any duplicate messages from individual users. The final processed dataset contains nearly 1 billion posts across 2.2 million unique accounts for all 104 weeks in 2019 and 2020 combined. At this point, 1418 counties (whose total population equals ~92% of the US population) are captured. Further statistics about the filtered CTLB are described in more detail in Table 1.

To maintain a minimum level of reliability for our depression and anxiety measurements users must post at least three times in a given week to be included in that week, and from our reliability testing we determined that counties must contain at least 200 unique users per week to be considered for any given week. The 3-user posting threshold (3-UPT) was determined to balance the diversity of users while minimizing noise from infrequent users. By applying a 3-UPT threshold there was a 37% decrease in unique user-week pairs, as opposed to a 23.4% decrease for 2-UPT and a 53% loss for 5-UPT thresholding. The 200-user threshold (UT) was determined by a reliability analysis whose results are shown in Fig. 2b. Counties that fail to report a score for 10 weeks consecutively are dropped from the dataset to remove the influence they pose to findings for a single week.

After applying our 3-UPT, 200-UT, and a max gap threshold of 10 (see Supplementary Fig. 4 for a robustness evaluation of this threshold), many posts belonging to mostly rural counties are necessarily excluded from our analysis. Since the target of this work is to better meet mental health reporting needs we implement a super-county binning strategy to reincorporate those “unreliable" county findings. All county-week findings that fail to meet the UT filter are weighted-mean aggregated by state into a super county-week result. Weights for the mean aggregation are assigned based on the reporting population of users of the included counties. Super counties must then pass the same UT set for regular counties to be included. In the case of UT = 200 this results in a gain of 9394 super county-week results over the original 35,353 county-week results. Figure 6b visually demonstrates how super-county binning reincorporates findings from unreliable counties.

The final post-processing step in our county-week pipeline is to run linear interpolation on a per county basis between missing weeks. For reference, at UT = 200 this translates to an increase from 35,288 to 35,969 county-weeks. When running our analyses in this work we opt to adjust 2020 county-week findings by removing periodicity effects by subtracting means for 2019. This adjustment highlights 2020-specific movement from week to week.

### Extract linguistic patterns

To extract language-based assessments of well-being from posts, we used existing lexical models of depression and anxiety^28,61^ that we adapted to 2019–2020 Twitter vocabularies using target-side domain adaptation^39^ which removes lexical signals that have different usage patterns (see target domain adaptation). The process for applying the model consists of extracting words from posts using the social media-aware tokenizer from the open-source Python package Differential Language Analysis ToolKit (DLATK; ^71^). Following^72^, the relative frequency of the words per user and unit of time is then Anscombe transformed to stabilize the variance of power law distribution. The approach then applies a linear model that is pretrained to produce anxiety and depression prediction scores from the word frequencies^28,73^. This produces a degree of depression (DEP_SCORE) and degree of anxiety (ANX_SCORE) for each user-time unit pair in the processed dataset, for this work that pair is user-week.

### Language-based depression and anxiety

The calculation of a language-based mental health assessment (*LBMHA*) centers on applying the pre-trained depression and anxiety weighted lexica^28,61^, *lex*_*s*_, following standard weighted lexicon scoring^74,75^:

1 $${L}_{s}({x}_{t})=\mathop{\sum}\limits_{w\,\in \,lex}{x}_{w}\times le{x}_{s}(w)$$

where *x*_*w*_ is the relative frequency of word, *w*, at the specific point in time, *t*, and *lex*_*s*_(*w*) is the word’s weight from the lexicon for *s* (depression or anxiety). Thus, *L*_*s*_ is a weighted sum of word frequencies. Because the original scales ranged from 0 to 5, lexicon scores were also thresholded to that range. It is important to note that while these lexica were previously validated in other contexts^28,61^, our study makes no assumption that they will generalize to this application, focusing on evaluating the aggregated county-week scores across reliability and validity criterion.

### Post-stratification and domain adaptation

Twitter is a biased sample of the American populace. Their users are more likely to be younger, and slightly more educated than the average American^76^. Further, the pre-trained lexica was derived using earlier social media language from Facebook. In order to correct for these discrepancies we apply both a validated post-stratified weighting scheme as well as domain adaptation.

We use robust post-stratification weights^29^, a pipeline for generating post-stratification weights, to compensate for under-representation in a dataset, from sparse and noisy data (i.e., demographic estimates from machine learning models applied to social media text). These weights allow us to aggregate biased samples to better represent the target populations being studied by removing selection biases (see Supplementary Fig. 5 for a robustness evaluation of these weights). Robust post-stratification first adjusts estimated demographic distributions to better match a target (estimator redistribution), then applies an adaptive binning process to make sure representation per demographic group is statistically powered. Finally, informed smoothing is applied from a known distribution of demographics to mitigate any remaining non-robust estimations^29^. In this work, robust post-stratification produces a weight per demographic bin of a particular Twitter account within a county, *ψ*(*x*_*t*_). This is then applied when taking a weighted mean of lexicon scores from accounts, *x*_*t*_ to a county, *c*, to produce the LBMHA:

2 $$LBMH{A}_{s}({c}_{t})=\frac{{\sum }_{x\in {c}_{t}}{L}_{s}({x}_{t})\times \psi ({x}_{t})}{{\sum }_{{x}_{t}\in {c}_{t}}\psi ({x}_{t})}$$

where *s* is the mental health lexicon (depression or anxiety) and *t* represents the particular time period (e.g., week).

### Target domain adaptation

The weighted anxiety and depression lexica were adapted to the specific target domain of 2019–2020 Twitter from the source domain of Facebook. These adaptations address drift in language usage and semantics from their source to the target following the *target-side domain adaptation* technique^39^. We adopted the domain adaptation criteria and applied it to filtering features and re-training the models, rather than applying it to set features to the mean. This process removed words from the original lexicon’s vocabulary, which contained 7680 unique words, that display different distributions in terms of either frequency or sparsity from their source^61,62^ to generate our domain-adapted well-being lexica^39^.

More precisely, domain usage outlier and relative frequency outlier filters were used to identify words that are used at different rates between the two domains of text. These outlier words are more likely to have significant differences in their semantics between domains^39,77,78^. As the correlation between the frequency of a word and outcomes for a given lexicon may differ for semantically different usages of a word, filtering words with different usages and frequencies limits our set of tokens to those that are more likely to carry similar cross-domain semantics (and thus, similar correlations).

The domain usage outlier filter was applied from *log*_10_ usage ratios *L*_*j*_ between [−1.0, 1.0] derived from Target relative word usage ${u}_{j}^{T}$ to Source relative word usage ${u}_{j}^{S}$. This left 6,214 remaining words and is computed as:

3 $${L}_{j}={\log }_{10}\left(\frac{{u}_{j}^{T}}{{u}_{j}^{S}}\right)$$

Then the relative frequency outlier filter was applied from frequency differences *d*_*j*_ between [−0.2, 0.2] from Target mean word frequency ${f}_{j}^{T}$ and Source mean word frequency ${f}_{j}^{S}$ scaled by the Source standard deviation of word frequency ${\sigma }_{j}^{S}$. This left 5765 remaining words and is computed as:

4 $${d}_{j}=\frac{{f}_{j}^{T}-{f}_{j}^{S}}{{\sigma }_{j}^{S}}$$

Additionally, words that are also common names according to the United States Social Security list (https://www.ssa.gov/oact/babynames/decades/) were dropped, yielding a final lexicon size of 5469.

Using DLATK ^71^, we created a weighted lexicon for both depression and anxiety by using the regression weights learned from retraining an existing ridge regression model to predict each outcome. A *k*-fold analysis determined that the best alpha to use on the ridge regression was 0.001, as well as preprocessing the data down to *k* = 500 components using principal component analysis. The final depression and anxiety lexica each contained 5469 word weights and an intercept weight.

### Reliability vs. resolution

At this point, we can begin to aggregate to a larger spatial or temporal resolution as necessary for analysis. To determine an appropriate resolution, we examine the finest resolution we can achieve while retaining reliable depression and anxiety score measurements.

To evaluate the reliability of a given spatio-temporal resolution, for each space-time pair in the resolution, we gather the set of users who posted at least three messages in this time period. If there are at least 20 such users, we randomly split the set into two approximately equally sized subsets and compute the split-half reliability (*R* = 1 − Cohen’s *d*) using their depression scores. Finally, the reliability is averaged across all space-time pairs.

Figure 2 shows the reliability scores of different spatiotemporal resolutions from running the procedure with counties in the New York City metropolitan area.

It is possible to generate reliable measures (*R* > 0.9) at the county-week level. We also analyze the effect of the threshold for the number of users per county-week pair on reliability. Figure 2 shows the reliability scores from running the aforementioned procedure with the entire CTLB data and with different thresholds for the number of users.

When relying on regional data, we report data that exceed a final group frequency threshold placed at 50 or 200 to match repeated split-half reliability (RSR) where RSR > 0.7, 0.8, and 0.9 for these thresholds respectively. *RSR* is calculated as the mean Cohen’s *d* of *N* repeated split-half samples into equal length *a* and *b* halves from the data belonging to a given region in time:

5 $$RSR=\frac{1}{N}\mathop{\sum }\limits_{i=1}^{N}1-\frac{{\mu }_{a}-{\mu }_{b}}{{\sigma }_{a\cup b}}$$

### Convergent validity

For Fig. 4 we look to the Gallup COVID-19 Panel^79^ to compare the validity of our measure and determine if these assessments are tracking the same underlying construct. Note that we do not treat the Gallup poll as a gold standard to exactly align with since the poll is a survey-based measure of self-reported sadness and worry, while our language based assessments are scores of depression and anxiety. The purpose of this particular study is to show a common alignment between a traditional survey method and an observational social media method. The Gallup data is based on individual responses to a survey which are then tagged with a week and a county of the respondent. This dataset covers 2617 counties with an average of ~4601 measurements per week across all counties. To this end, we use fixed effect multi-level modeling to remove the effects of endogeneity bias stemming from inherent between-county differences. While LBMHA scores are already held to a baseline 1-Cohen’s *d* reliability of 0.9, Gallup results are held to a standard of 0.7. If this adjustment is not made there are no counties collected by Gallup for which county-week results are reliable for the full 22 weeks the survey covered.

### External criteria

To compare our assessments cross-sectionally against other external measurements we look to the County Health Rankings (CHR)^36^. From CHR 2020 we look to political, economic, social, and health-based outcomes at the county level. For political variables, we evaluate the proportion of county voters who voted Republican in 2016 and 2020 and Third party in 2020. For economic variables, the logged median household income, the unemployment rate, and the proportion of people over age 24 holding bachelor’s degrees. For social variables, the per capita number of social associations, the violent crime rate, and the percent of youth unaffiliated with school or a similar organization. For health variables, the surveyed percent of people reporting fair or poor health, the age-adjusted suicide rate, and the age-adjusted mortality rate. LBMHAs were limited to the same cross-sectional period as was covered by the Gallup survey and reported correlations controlled for geographic effects at the state level. Figure 2b extends the cross-sectional test of validity to conduct a longitudinal study of major events on measurements across counties. For this work, we examine the weekly changes in county measurements of anxiety and depression during weeks where major US events occurred and weeks where they did not occur. Combining 14 events identified by The Uproar^80^ with 18 events from Business Insider^81^ we arrived at 14 weeks in 2020 as “major US event weeks” (13 events were in common between the news sources and a single week could contain more than 1 event). These events were chosen a priori to prevent specific event choices as parameters to the test. It was not clear that the scale of events could be captured, without introducing potentially confounding social media impressions, so we used the events chosen by the articles. We then filtered these events to those that happened within the United States (including those applying globally, such as pandemic onset) arriving at 14 total event weeks to compare with 38 non-event weeks. An event week is defined as an ISO week which contains the date any of the labeled major events occurred. A 1 day buffer is added to the date of the event before mapping to a week so that scoring changes caused by the event can be captured. For each sample of event and non-event weeks, we collect the percent change in national-week depression and anxiety scores from the previous week. Using these two samples we compute Cohen’s *d* between the event week and non-event week findings. To establish a confidence interval we use Monte Carlo bootstrapping over 10,000 iterations of event and non-event weeks.

### Inclusion and ethics

All procedures were approved by the Stony Brook University Institutional Review Board. The review board found the study to be exempt due to not pertaining to human subjects research. We have no human subject participants from an ethics guidelines perspective due to using only public, pre-existing data. Further details can be found in IRB2020-00587 Social Media for Aggregate Mental Health Monitoring.

### Reporting summary

Further information on research design is available in the Nature Research Reporting Summary linked to this article.

### Supplementary information

Supplementary Information

Reporting Summary

## Data Availability

The dataset of county-week LBMHAs generated and analyzed during the current study is available in the WWBP Github repository, github.com/wwbp/lbmha_2019-2020.

## Code availability

To support open science, we provide an open-source toolkit to run the LBMHA pipeline as well as data describing the results per county week. The pipeline and relevant analysis scripts will be available at github.com/wwbp/robust_spatiotemp. Additional code used for generating robust post-stratified weights can be found at github.com/wwbp/robust-poststratification.
