# Supplementary material for: Robust language-based mental health assessments in time and space through social media
Source: NPJ Digit Med. 2024 May 2;7:109. doi: 10.1038/s41746-024-01100-0 (PMC11065872; doi:10.1038/s41746-024-01100-0)
Supplement: Supplementary file 1 — Supplementary Information [file 41746_2024_1100_MOESM1_ESM.pdf]

Supplementary Information

Supplementary Figures

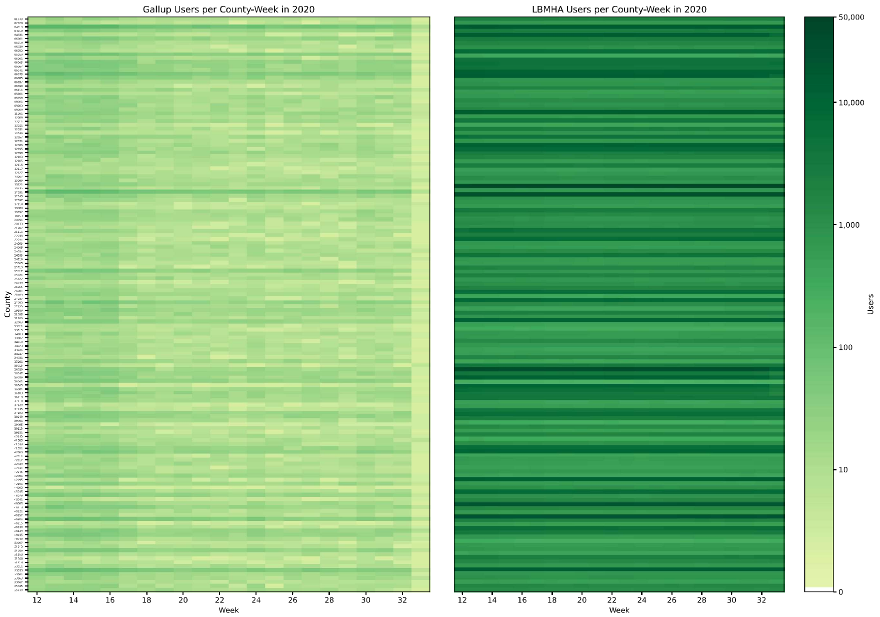

Supplementary Figure 1: Visualization of the data that is used in our fixed effects analysis. This graphic only includes matching LBMHA and Gallup counties for which we have reliable LBMHA measurements, i.e. LBMHA county assessments with at least 200 unique users in that week and that pass the max gap threshold. Additionally, to be used in this fixed effects analysis and appear in this diagram the county must not be missing any data for the 22 weeks studied.

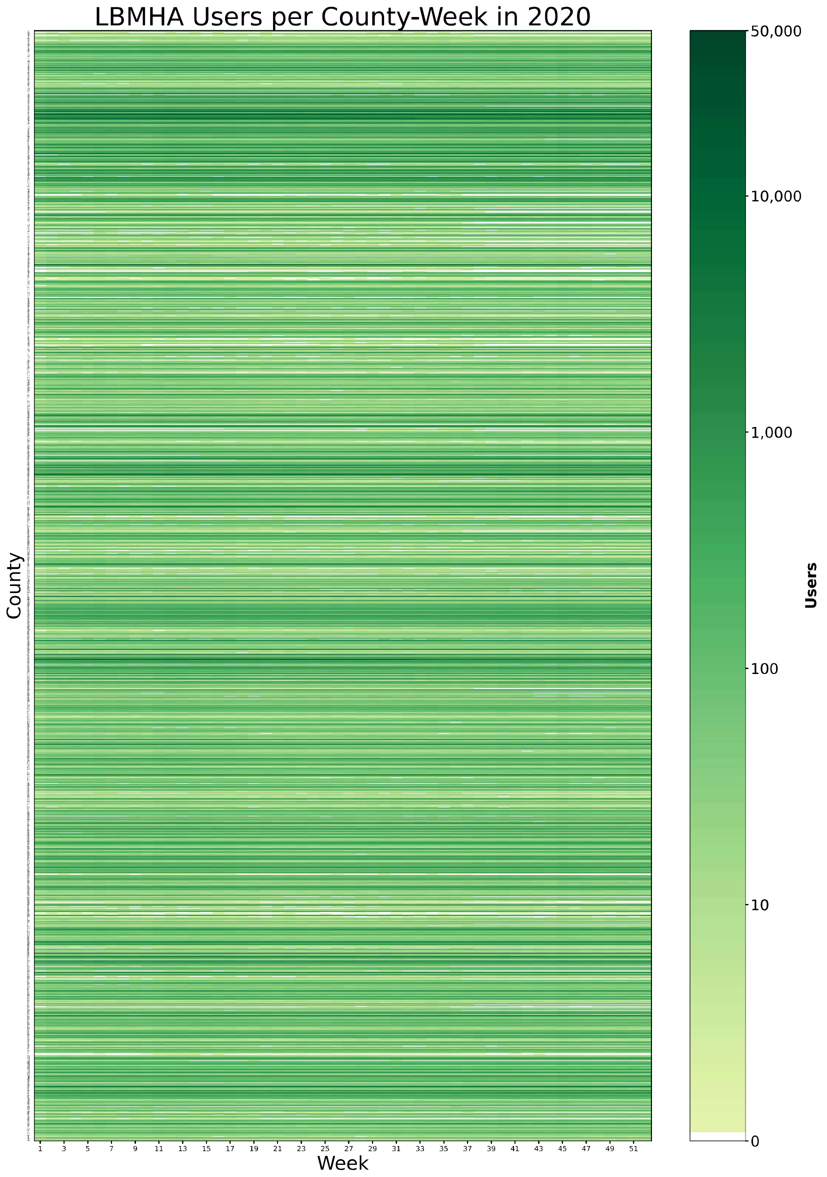

Supplementary Figure 2: Visualization of the data coverage for all counties in our 2020 dataset controlling for the previous year. This graphic does not include our interpolation, UT, or max gap filtering.

		Without Post-Strat		With Post-Strat	
		**Depression**	**Anxiety**	**Depression**	**Anxiety**
**Space** (N)	**Time** (N)	β	β	β	β
National (1)	Weeks (22)	0.754†	1.760‡	0.763†	1.823‡
Regions (4)	Weeks (22)	0.761‡	1.763‡	0.759‡	1.817‡
Counties (132)	Quarters (3)	0.865‡	1.504‡	0.681‡	1.423‡
Counties (132)	Weeks (22)	0.444‡	0.300‡	0.410‡	0.343‡

Supplementary Figure 3: Robustness Analysis of Convergent Validity based on Post-stratification. Fixed effects coefficients without and with post-stratification. Dropping post-stratification had no change to the conclusions drawn on convergent validity, although effect-sizes without post-stratification were smaller for the national-week measurements, suggesting mitigating the selection bias can improve convergence. Representation disparity went from 9.77 to 9.43 where disparity is measured as the mean absolute error (MAE) between the observed per county demographics bins in CTLB2 before and after applying weights and 2020 census bins. Bins here are the percentage of individuals with incomes $\geq$\$0, $\geq$\$10,000, $\geq$\$15,000, $\geq$\$25,000, $\geq$\$35,000, $\geq$\$50,000, $\geq$\$75,000, $\geq$\$100,000, $\geq$\$150,000, and $\geq$\$200,000. This represented a 6.5% mean non-zero improvement in MAE-based census disparity reduction. Results significant at: $^{\ddagger}p < .001$, $^{\dagger}p < .01$

	**Anxiety**	
MGT	**20**	**10**
**5**	0.9989	0.9995
**10**	0.9995	

	**Depression**	
MGT	**20**	**10**
**5**	0.9996	0.9998
**10**	0.9999	

Supplementary Figure 4: Robustness Analysis of convergent validity based on the max gap thresholds (MGT). The main results use an MGT of 10. Here we show the correlations of the nation-week measurements (i.e. the data behind Figure 4b upon varying the MGT.

	Moran's I Mean (S.D.)
Depression	$0.216\ (0.0788)^{\ddagger}$
Anxiety	$0.290\ (0.121)^{\ddagger}$

(a) Moran's I results

**Space** (N)	**Time** (N)	**Depression** β	**Anxiety** β
National (1)	Weeks (22)	$0.592^{\dagger}$	$0.748^{\dagger}$
Counties (132)	Weeks (22)	$0.390^{\ddagger}$	0.318^{*}

(b) Results after controlling for spatial autocorrelation

Supplementary Figure 5: Robustness Analysis of convergent validity to spatial auto-correlation. Fixed-effects above are controlled for spatial auto-correlation of neighboring counties in (a). All convergence results remained significant while the fixed effects coefficients reduced a small amount as compared to results when not controlled for spatial autocorrelation. As seen in (b) The average Moran's I value across all county-weeks was 0.216 for depression and 0.290 for anxiety. This indicates a low to moderate amount of spatial auto-correlation demonstrating greater heterogeneity from one county to it's neighbors than for example previous measures of personality by language [84].
Results significant at: $^{\ddagger}p < .001$, $^{\dagger}p < .01$, $^{*}p < .05$

	Sad / Depression		Worry / Anxiety	
	**F1**	**AUC**	**F1**	**AUC**
**High / Low**	0.6364	0.8264	0.7273	0.7851
**High / Low (ternary)**	0.7059	0.8571	0.8000	0.875

Supplementary Figure 6: Accuracy metrics when recasting our fixed effects analysis as a classification task rather than regression. We report F1 Macro and AUC scores for National-Week measurements between Gallup and LBMHAs. Here we use the same data from our analysis in Figure 4a. In the first High/Low task the results for both LBMHA and Gallup are independently binned into High and Low results relative to their respective medians. In the second High/Low (ternary) task Gallup is instead split into High/Medium/Low and all Medium results are dropped before calculating F1s and AUCs, this captures the ability of LBMHAs to capture meaningful changes above and below the median.
